# Metabolic reprogramming in chondrocytes to promote mitochondrial respiration reduces downstream features of osteoarthritis

**DOI:** 10.1038/s41598-021-94611-9

**Published:** 2021-07-23

**Authors:** Yoshifumi Ohashi, Nobunori Takahashi, Kenya Terabe, Saho Tsuchiya, Toshihisa Kojima, Cheryl B. Knudson, Warren Knudson, Shiro Imagama

**Affiliations:** 1 Department of Orthopedic Surgery, Nagoya University Graduate School of Medicine, 65 Tsurumai-cho, Showa-ku, Nagoya, 466-8550 Japan; 2 Department of Orthopedic Surgery, Japan Community Health Care Organization, Tokyo Shinjuku Medical Center, 5-1 Tsukudo-cho, Shinjuku-ku, Tokyo, 1628543 Japan; 3 Department of Anatomy and Cell Biology, Brody School of Medicine, East Carolina University, Greenville, NC 27834 USA

**Keywords:** Cell biology, Diseases, Molecular medicine, Risk factors

## Abstract

Metabolic dysfunction in chondrocytes drives the pro-catabolic phenotype associated with osteoarthritic cartilage. In this study, substitution of galactose for glucose in culture media was used to promote a renewed dependence on mitochondrial respiration and oxidative phosphorylation. Galactose replacement alone blocked enhanced usage of the glycolysis pathway by IL1β-activated chondrocytes as detected by real-time changes in the rates of proton acidification of the medium and changes in oxygen consumption. The change in mitochondrial activity due to galactose was visualized as a rescue of mitochondrial membrane potential but not an alteration in the number of mitochondria. Galactose-replacement reversed other markers of dysfunctional mitochondrial metabolism, including blocking the production of reactive oxygen species, nitric oxide, and the synthesis of inducible nitric oxide synthase. Of more clinical relevance, galactose-substitution blocked downstream functional features associated with osteoarthritis, including enhanced levels of MMP13 mRNA, MMP13 protein, and the degradative loss of proteoglycan from intact cartilage explants. Blocking baseline and IL1β-enhanced MMP13 by galactose-replacement in human osteoarthritic chondrocyte cultures inversely paralleled increases in markers associated with mitochondrial recovery, phospho-AMPK, and PGC1α. Comparisons were made between galactose replacement and the glycolysis inhibitor 2-deoxyglucose. Targeting intermediary metabolism may provide a novel approach to osteoarthritis care.

## Introduction

Previous studies have suggested that the degradative features associated with osteoarthritis (OA) in articular cartilage are the downstream result of changes in the metabolism of resident chondrocytes^1,2^. These OA features include enhanced production of extracellular proteinases such as MMPs, ADAMTS4/5, the resultant loss of proteoglycan, and eventual breakdown of the collagenous network. Changes in metabolism associated with OA chondrocytes include enhanced dependence on glycolysis for cellular ATP production, coordinate with a decrease in mitochondrial respiration, and use of the TCA cycle^2^. This alteration has many features in common with the Warburg Effect that proposed that mitochondrial defects are the underlying basis for aerobic glycolysis and cancer^3^ seen in cancers and proliferating healthy cells.

We have recently gained insight into the features of these metabolic changes in chondrocytes and how they can be reversed^1,2^. Initially, we observed that enhanced local synthesis of hyaluronan (HA) via viral overexpression of HA synthase-2 (HAS2-OE) blocked and reversed the pro-catabolic features of normal chondrocytes activated by IL1β, TNFα or LPS as well as human OA-derived chondrocytes^1^. Surprisingly, however, this was not due to enhanced accumulation of extracellular HA. Rather, viral driven enhancement of HA polysaccharide synthesis altered the intermediary metabolism of the activated chondrocytes due to over-usage of intracellular UDP-sugar precursor pools. Similar results were obtained by the treatment of chondrocytes with 4-methylumbelliferone (4MU), a known inhibitor of HA that acts in part by sequestration and depletion of UDP-glucuronic acid^4^. HAS2-OE and 4MU both cause a reduction in the enhanced usage of the glycolysis pathway by activated, pro-catabolic chondrocytes. The HAS2-OE chondroprotective effect can also be mimicked by treatment of activated chondrocytes with 2-deoxy-glucose (2DG), which is a direct glycolysis inhibitor. Interestingly, in all these treatments, not only was glycolysis reduced, but ATP production from oxidative phosphorylation (OXPHOS) was enhanced. This paper seeks to explore how these two metabolic pathways are coupled and which events are critical to the chondroprotective effects.

This study investigated the effects of total glucose replacement with the monosaccharide isomer galactose. Galactose substitution is often used in studies on the downstream effects of metabolic dysfunction in cancer or normal proliferating cells. Galactose substitution is a known activator of OXPHOS coupled with suppression of glycolysis for the production of ATP. Galactose is metabolized via the Leloir pathway^5–7^ with the conversion of galactose into G6P, which then can be used for the glycolysis pathway. Typically, however, the conversion of galactose to G6P is slower than the rate by which glucose becomes G6P^6^. Therefore, the production of pyruvate via glycolytic metabolism of galactose produces lower amounts of ATP. This forces the cells to use an increased reliance on ATP production from OXPHOS. This, in turn, induces a strong compensatory activation of OXPHOS, at least in cells that retain the capacity to adapt and rescue mitochondrial function. For example, healthy cells adapt and survive in either glucose or galactose culture media, whereas many tumor cells with impaired mitochondrial function fail to survive^8^. Galactose substitution also provides an improved approach to complete or partial shutdown of glycolysis by glucose deprivation alone—a condition that often induces cell death^9^.

## Results

### Galactose suppresses glycolysis and enhances mitochondrial respiration

**Figure 1:**
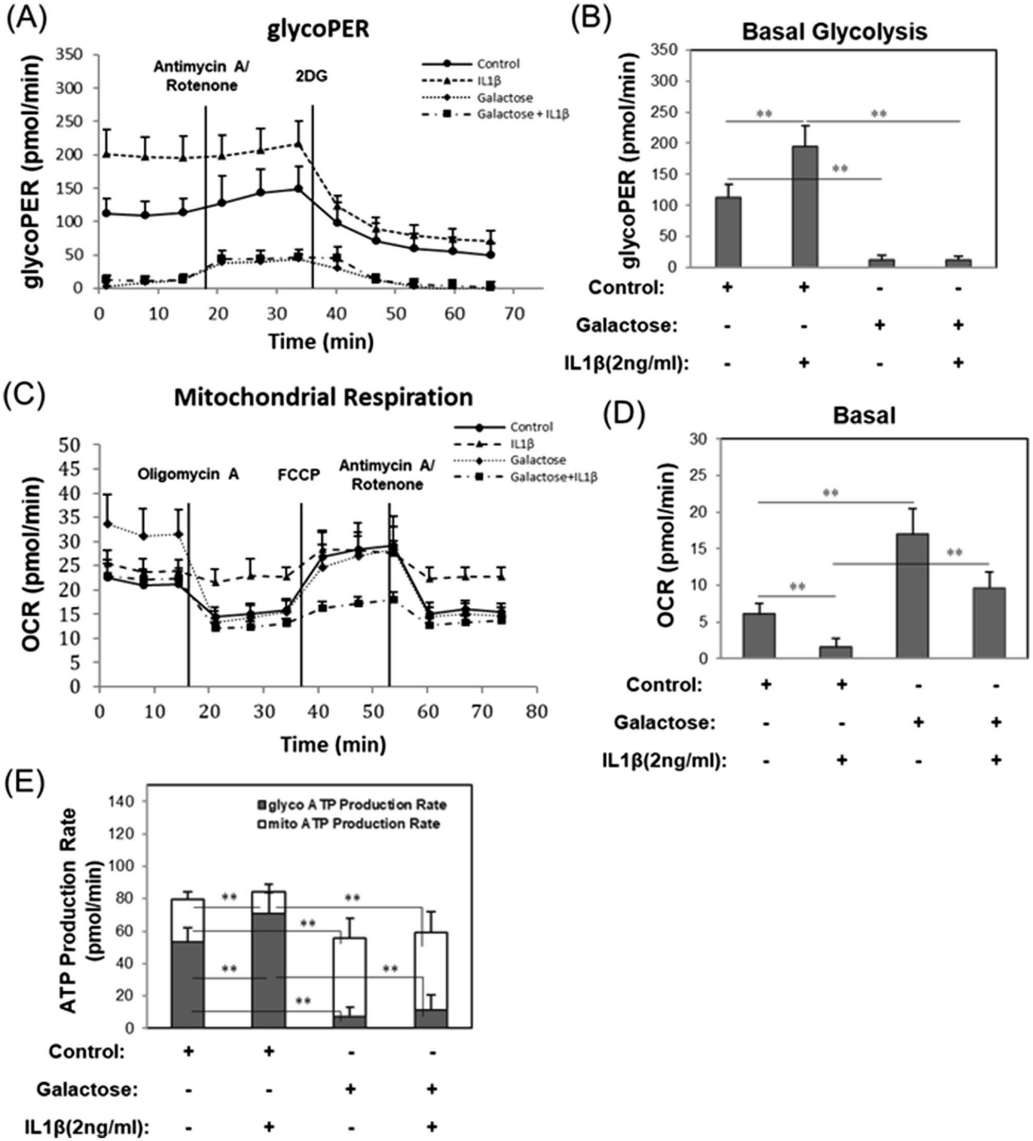
Galactose suppresses glycolysis and enhances mitochondrial respiration. (**A**) Representative Glycolytic Rate Assay kinetic plot wherein values for proton efflux rate (PER, pmol/min) are plotted versus time. Note that rotenone and antimycin A were injected at 18 min followed by high dose 2DG injection at 36 min. (**B**) Summaries of PER data representative of changes in basal glycolysis rates as labeled [mean ± S.D. (error bars), n = 10]. (**C**) Representative Mito Stress Test kinetic plot wherein values for Oxygen Consumption Rate (OCR, pmol/min) are plotted versus time. Note that oligomycin A was injected at 18 min followed by FCCP injection at 36 min. rotenone and antimycin A were injected at 54 min. (**D**) Summaries of OCR data representative of changes in basal mitochondrial respiration as labeled [mean ± S.D. (error bars), n = 8]. (**E**) Representative Real-Time ATP Production Rate Assay in bovine chondrocytes wherein the contribution of glycolysis (gray bars) and mitochondrial respiration (open bars) to ATP production after treatments for 24 h are as labeled [mean ± S.D. (error bars), n = 10]. ANOVA followed by Tukey post-hoc test was used for statistical analysis. *p < 0.05; **p < 0.01.

Bovine chondrocytes were analyzed in real time for changes in rate of H^+^ proton efflux into the culture medium (PER) indicative of glycolytic metabolism. Raw data in Fig. 1A shows changes in PER for each culture condition, that occur following injections of select OXPHOS inhibitors: Antimycin A/Rotenone, and a glycolysis inhibitor: 2DG at 18 and 36 min during the rate assay—inhibitors that define the pathways responsible for the proton release. Calculations were made from such plots from multiple experiments to quantify changes in basal glycolysis (Fig. 1B). Following treatment with IL1β, PER became substantially elevated (Fig. 1A,B). However, if chondrocytes were cultured in galactose-replaced medium, this elevation was not only blocked but PER was reduced to levels lower than control cells. Using a mitochondrial stress assay, real time changes in oxygen consumption rate (OCR) were obtained (Fig. 1C,D). Raw data (Fig. 1C) include injections of OXPHOS inhibitors: Oligomycin A, carbonyl cyanide-4-phenylhydrazone (FCCP), and Antimycin A/Rotenone at 18, 36, and 54 min were used to define mitochondrial contribution to OCR; with data summarized in Fig. 1D. Treatment of chondrocytes with IL1β resulted in a prominent reduction in mitochondrial respiration as compared to control chondrocytes (Fig. 1C,D). Culture of chondrocytes treated with IL1β in galactose-replaced medium did not exhibit a diminution of OCR. Moreover, OCR levels of chondrocytes in galactose-replaced medium in the absence of IL1β were significantly enhanced as compared to control chondrocytes in glucose-enriched medium.

Another test performed using the Seahorse flux analyzer, termed a Real-Time ATP rate assay, generated similar PER and OCR data to define the pathway contributions to ATP production in (pmol/min ATP). In these assays the contribution of ATP derived from OXPHOS is determined in part by the difference between basal OCR and OCR levels following injection of Oligomycin A, a potent inhibitor of mitochondrial ATP synthase. These data are summarized in the stacked bar plot shown in Fig. 1E. Control chondrocytes utilized glycolysis to generate approximately 75% of their ATP (Fig. 1E, grey bar); with 25% obtained from OXPHOS (Fig. 1E, white bar). After treatment with IL1β, chondrocytes became more dependent on glycolysis as the source of cellular ATP, with only a small percentage coming from OXPHOS. However, when these same conditions were analyzed in a galactose-replaced medium the metabolic changes due to IL1β were blocked. Interestingly, under the galactose-replacement conditions, ATP production due to OXPHOS, in both control and IL1β treated cultures, was enhanced with glycolysis now providing only a minor contribution.

### IL1β, 2DG, or Galactose culture conditions do not cause changes in cell proliferation of primary chondrocytes

**Figure 2:**
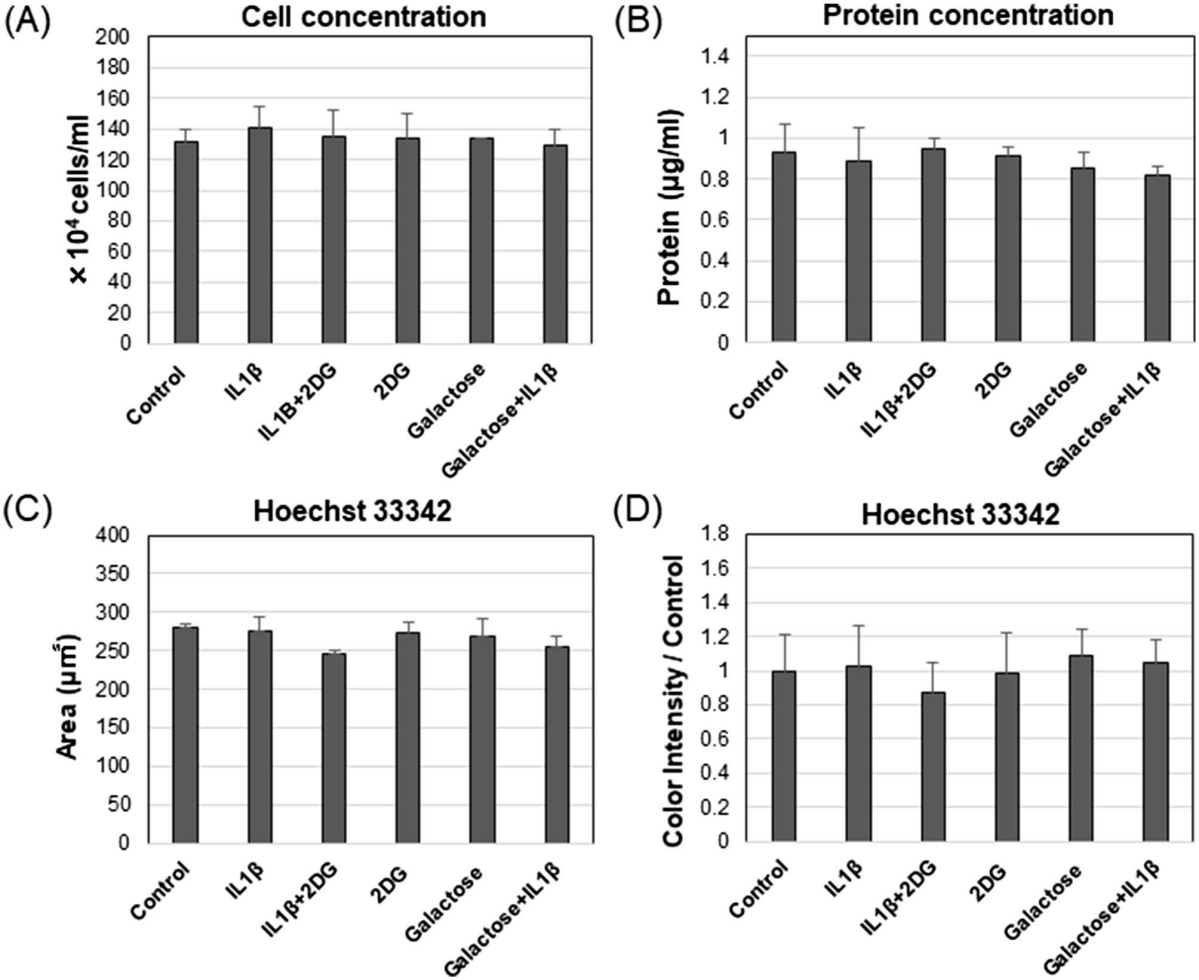
IL1β, 2DG, or galactose culture conditions did not cause any changes to cell proliferation of primary chondrocytes. (**A**) Bar graph of cell number comparing each condition [mean ± S.D. (error bars), n = 4]. (**B**) Bar graph of protein concentration comparing each condition [mean ± S.D. (error bars), n = 4]. (**C**) Bar graph of the total Hoechst 33342-stained cell occupied area [mean ± S.D. (error bars), n = 3]. (**D**) Bar graph of the color intensity of Hoechst 33342 [mean ± S.D. (error bars), n = 3] ANOVA followed by Tukey post-hoc test was used for statistical analysis. *p < 0.05; **p < 0.01. Representative images of brightfield and fluoroscopy are shown in Supplementary Fig. S5.

To accurately determine whether any of the reagents (IL1β and 2DG) or cell culture conditions (glucose and galactose culture) used in this study resulted in potential changes in cell number, chondrocytes were plated into six well plates and treated as in experiments shown in Fig. 1. By automated cell counting, no difference in cell numbers were observed between control and each test condition (Fig. 2A). BCA protein determination assays of isolated cell lysates revealed no significant differences in total protein (µg/ml) between control cultures and each experimental condition (Fig. 2B). When chondrocytes were stained with the DNA labeling dye Hoechst 33342, no differences were observed in total stained cell-occupied-area (Figs. 2C and S5) or color intensity (Figs. 2D and S5) between each condition as quantified using fluorescent image analysis software. Thus, there appears to be no evidence that the culture reagents or conditions exerted a toxic or proliferative effect on monolayer cultures of bovine chondrocytes.

### Effects of IL1β and galactose mitochondrial integrity and function

**Figure 3:**
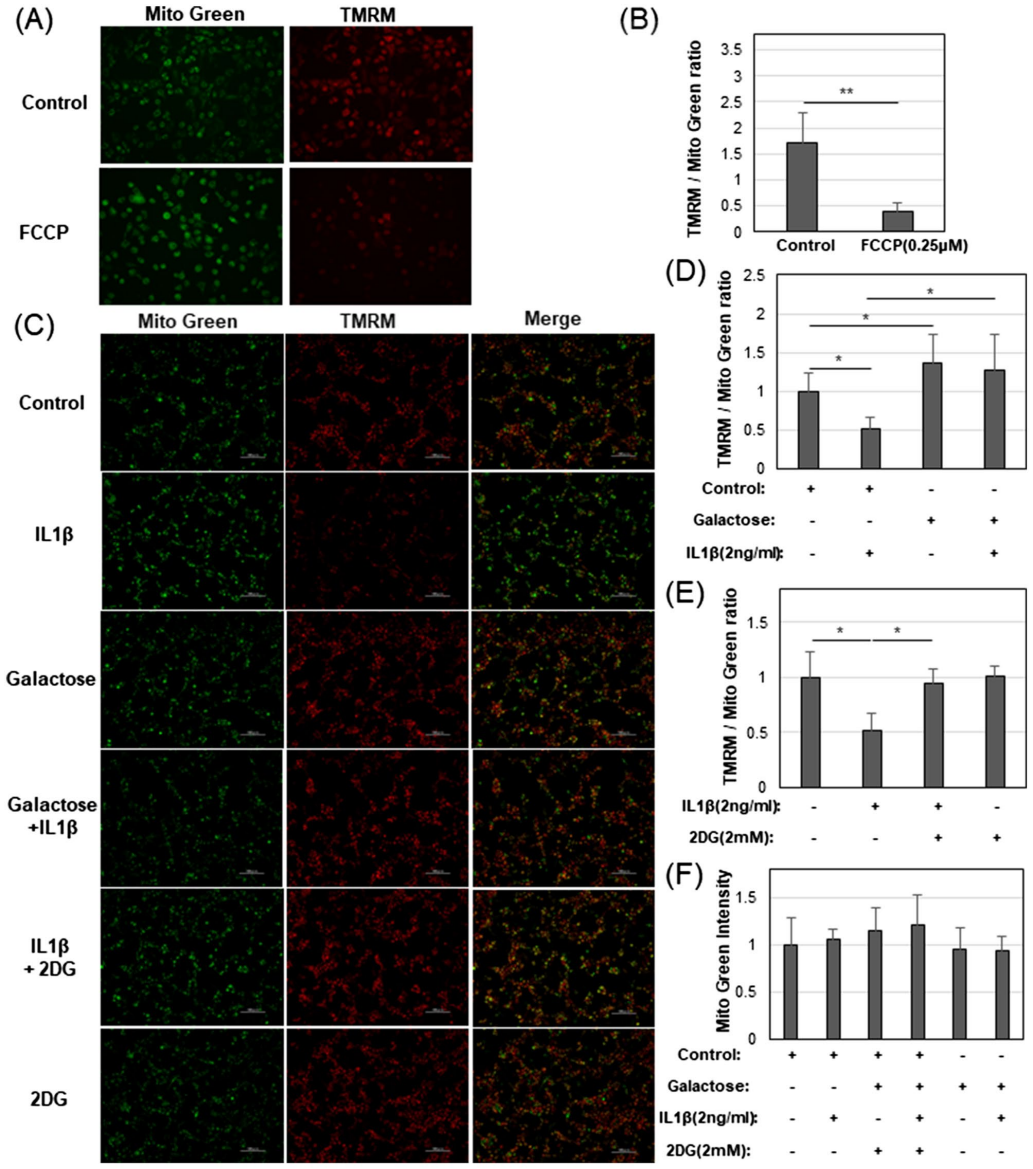
Effects of IL1β and galactose on mitochondrial integrity and function. (**A**) Representative images of Mito Green and TMRM stained chondrocytes in Control glucose medium without or with FCCP. (**B**) Bar graph of TMRM/Mito Green ratio without or with FCCP. (**C**) Representative images of Mito Green, TMRM, and merge in Control glucose or galactose medium with or without IL1β and 2DG as labeled. (**D**) Bar graph of TMRM/Mito Green ratio of Control, IL1β, Galactose, Galactose + IL1β culture conditions. (**E**) Bar graph of TMRM/Mito Green ratio from Control glucose, IL1β, IL1β + 2DG, and 2DG culture conditions. (**F**) Bar graph of the color intensity of Mito Green alone in the conditions labeled. Displayed images (**A**, **C**) are digitally enhanced to 40% brighter and processed by haze reduction. The color intensity (**B**, **D**–**F**) was calculated from raw images and bar graphs show the average ± S.D. (error bars), n = 6 experiments. Student’s t-test was used for statistical analysis in panel B. ANOVA followed by Tukey post-hoc test was used for statistical analysis in (**D**–**F**). *p < 0.05; **p < 0.01.

Given the effects of IL1β and galactose on mitochondrial respiration and OXPHOS in chondrocytes, we next determined whether this was due to an increase in the number of mitochondria or their functional integrity. The red fluorescent Tetramethylrhodamine (TMRM) reagent accumulates in active mitochondria with an intact membrane potential whereas the reagent *Mito Tracker Green FM* reagent (Mito Green) reacts with free cysteine thiol groups of mitochondrial proteins and identifies all mitochondria. Stained control chondrocytes exhibited close overlap of green and red fluorescence suggesting that nearly all mitochondria were active. When the cells were treated with an inhibitor of oxidative phosphorylation, FCCP, the mitochondrial membrane potential collapsed by uncoupling electron transport from ATP generation, TMRM became diffusely distributed throughout the cytosol while the green fluorescence remained intact (Fig. 3A). A ratio of TMRM/Mito Green fluorescence intensity was used to quantify these changes (Fig. 3B).

Chondrocytes incubated with IL1β readily lost significant levels of TMRM fluorescence as compared to control chondrocytes (Fig. 3C). However, in chondrocytes cultured in galactose-replaced medium, the IL1β-induced reduction in TMRM accumulation was blocked (Fig. 3C,D). Moreover, the TMRM/Mito Green ratios of chondrocytes in galactose-replaced medium were higher than those of control chondrocytes (Fig. 3C,D). A similar observation was made when chondrocytes were treated with 2DG. 2DG (in glucose-containing medium) also blocked the accumulation of TMRM following treatment with IL1β (Fig. 3C,E). However, with the addition of 2DG, the TMRM/Mito Green ratios remained equivalent to control chondrocytes.

When Mito Green fluorescence was quantified separately, no significant changes were observed under any test conditions of culture (Fig. 3F) suggesting that changes in mitochondrial respiration and OXPHOS shown in Fig. 1 were not due to changes in the number of mitochondria per cell.

### Galactose blocks markers of mitochondrial damage

**Figure 4:**
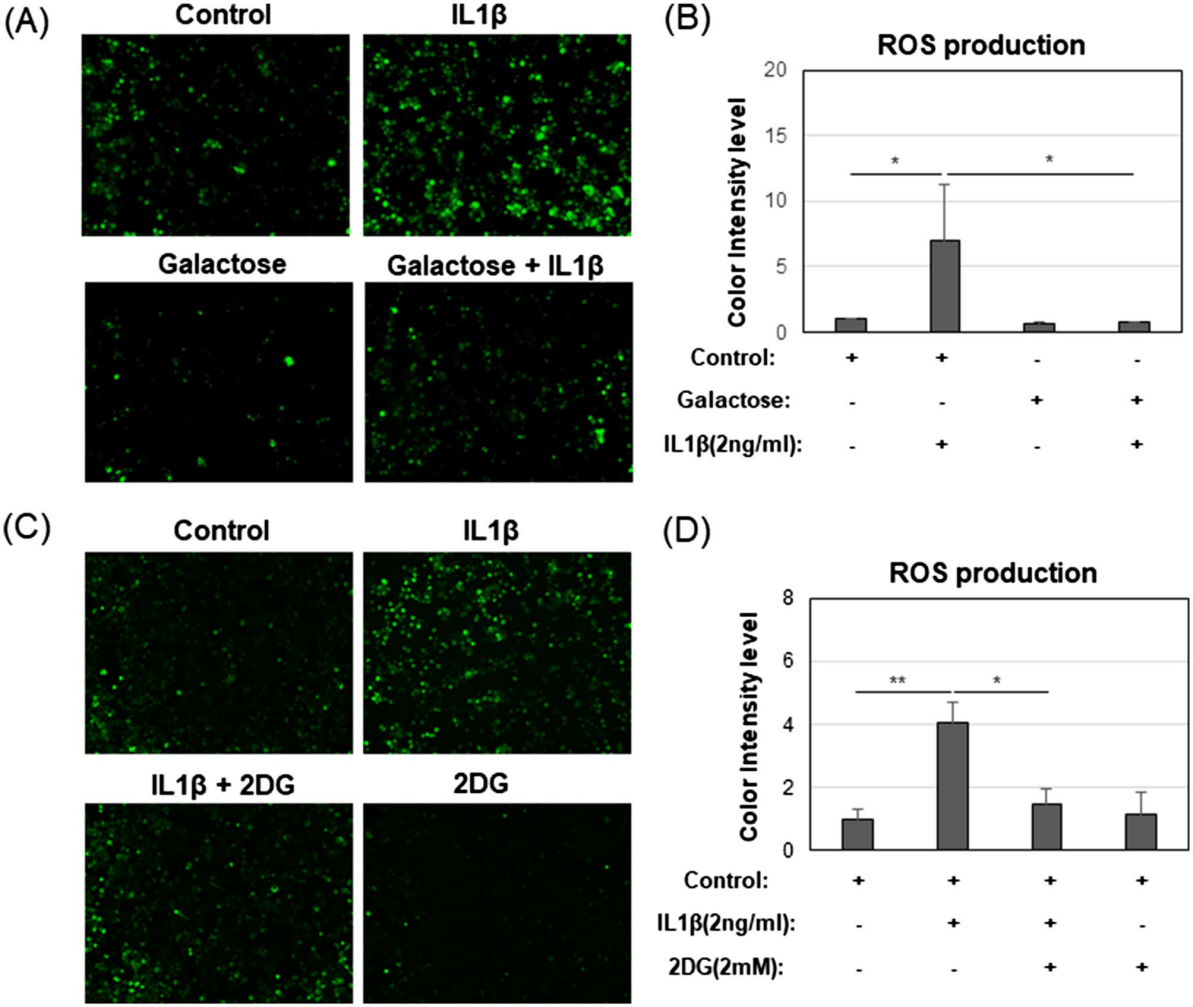
Galactose blocks ROS markers of mitochondrial damage. (**A**) Representative ROS Assay images for Control, IL1β, Galactose, and Galactose + IL1β culture conditions. (**B**) The quantified color intensity from (**A**) [mean ± S.D. (error bars), n = 3]. (**C**) Representative ROS Assay images for Control, IL1β, IL1β + 2DG, and 2DG. (**D**) The quantified color intensity from (**C**) [mean ± S.D. (error bars), n = 3]. ANOVA followed by Tukey post-hoc test was used for statistical analysis. *p < 0.05; **p < 0.01.

Reactive oxygen species (ROS) are well known by-products of damaged mitochondria, especially in pro-catabolically activated chondrocytes^9^. ROS were detected in live cells using a cell-permeant green fluorescent reagent, 2,7–dichlorofluorescein diacetate (DCFDA). In control chondrocytes, a few green fluorescent-positive cells were observed scattered throughout various fields of view (Fig. 4A); intensity values quantified in Fig. 4B. As expected, treatment with IL1β dramatically increased the number of green fluorescent-positive cells. This increase in ROS was significantly blocked in chondrocytes treated with IL1β under galactose-replaced medium culture conditions (Fig. 4A,B). These results were compared to a second series of experiments where chondrocytes were treated with or without IL1β and, with or without 2DG (Fig. 4C,D). Again, IL1β treatment caused a pronounced increase in ROS that was blocked by the presence of 2DG.

**Figure 5:**
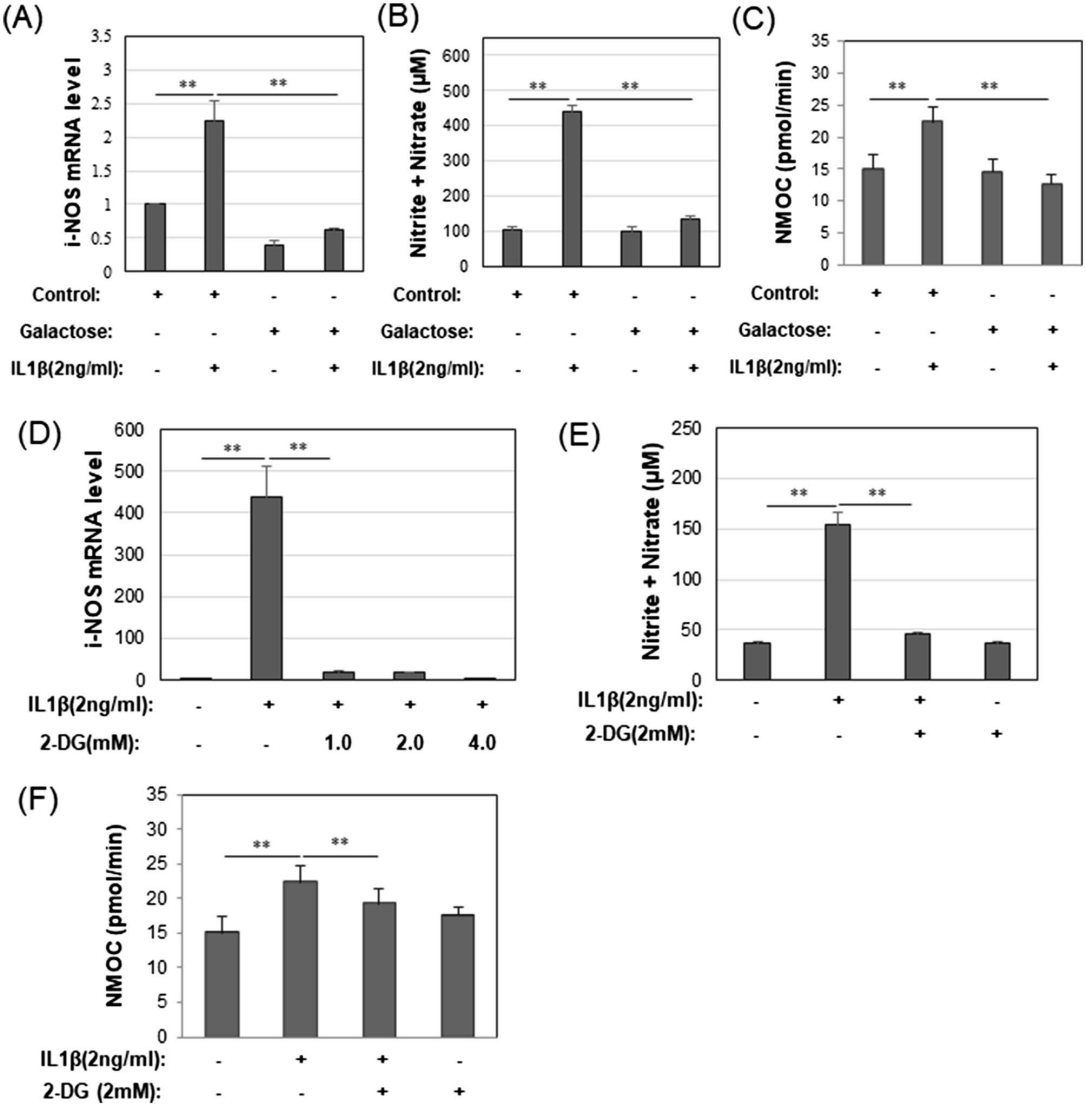
Galactose blocks the end products of NO and NMOC markers of mitochondrial damage. (**A**) The mRNA of inducible nitric oxide synthase (iNOS) was measured by RT-Real Time PCR for chondrocytes in Control glucose or galactose culture condition treated with or without IL1β [mean ± S.D. (error bars), n = 3]. (**B**) Total amount of Nitrite and Nitrate in cell culture medium was determined for chondrocytes in Control glucose or galactose culture condition treated with or without IL1β [mean ± S.D. (error bars), n = 3]. (**C**) Non mitochondrial Oxygen Consumption Rate (OCR) by chondrocytes treated with or without IL1β in Control glucose or galactose cultures as labeled [mean ± S.D. (error bars), n = 8]. (**D**) mRNA levels of inducible nitric oxide synthase (iNOS) were measured by qRT-Real Time PCR for chondrocytes in Control glucose conditions treated with or without IL1β and 2DG [mean ± S.D. (error bars), n = 3]. (**E**) NO (total amount of Nitrite and Nitrate) in cell culture medium was determined for chondrocytes in Control glucose culture conditions treated with or without IL1β and 2DG [mean ± S.D. (error bars), n = 3]. (**F**) Non mitochondrial Oxygen Consumption Rate (OCR) by chondrocytes treated with or without IL1β and 2DG in Control Glucose cultures as labeled [mean ± S.D. (error bars), n = 8]. ANOVA followed by Tukey post-hoc test was used for statistical analysis. *p < 0.05; **p < 0.01.

Nitric oxide (NO) is another oxygen species that is altered in OA and pro-catabolically activated chondrocytes and; is one of the causes of damage to the mitochondrial electron transport system^10^. First, the expression of inducible nitric oxide synthase (iNOS) mRNA was examined by qRT-PCR. As expected, IL1β increased of iNOS mRNA relative to the expression in control chondrocytes (Fig. 5A). However, when chondrocytes were grown under galactose-replaced medium conditions, the increase in iNOS mRNA expression was blocked and, reduced below control baseline levels. iNOS mRNA levels in control chondrocytes grown in galactose-replaced medium were also ~ twofold lower than control chondrocytes grown in glucose-rich medium. In a separate series of experiments, the glycolysis inhibitor, 2DG also blocked IL1β induced increases in iNOS mRNA (Fig. 5D). Changes in the amount of Nitrite and Nitrate, that are inert end products of NO metabolism, were also monitored (Fig. 5B). Following the trend in iNOS mRNA, treatment of chondrocytes with IL1β generated a pronounced increase in the amount of Nitrite and Nitrate as compared to control chondrocytes (Fig. 5B). This IL1β-induced increase in the amount of Nitrite and Nitrate did not occur when the chondrocytes were cultured under galactose-replaced medium conditions. Again, in a separate series of experiments, the glycolysis inhibitor, 2DG also blocked IL1β induced increases in iNOS and the amount of Nitrite and Nitrate (Fig. 5D,E).

As a third approach, the Seahorse flux analyzer was used to measure and quantify in real-time non-mitochondrial oxygen consumption (NMOC); representing oxygen consumption that contributes to the production of both NO and ROS^9,11^. NMOC is measured after completely blocking mitochondrial respiration using a combination of OXPHOS inhibitors: Oligomycin A, FCCP, and Antimycin A/Rotenone. In glucose-rich medium conditions, IL1β treatment resulted in an increase in NMOC-related OCR (oxygen consumption rate) above that of control chondrocytes (Fig. 5C). However, in galactose-replaced medium this increase in NMOC-related OCR was blocked. Treatment of chondrocytes with IL1β in the presence of 2DG (in glucose-containing medium) resulted in a similar block of IL1β-induced increase (Fig. 5F).

These results suggest that galactose-replaced medium blocks the production and accumulation of oxygen by-products highly associated with mitochondrial damage.

### Galactose-replaced culture conditions block pro-catabolic features of activated chondrocytes and cartilage

**Figure 6:**
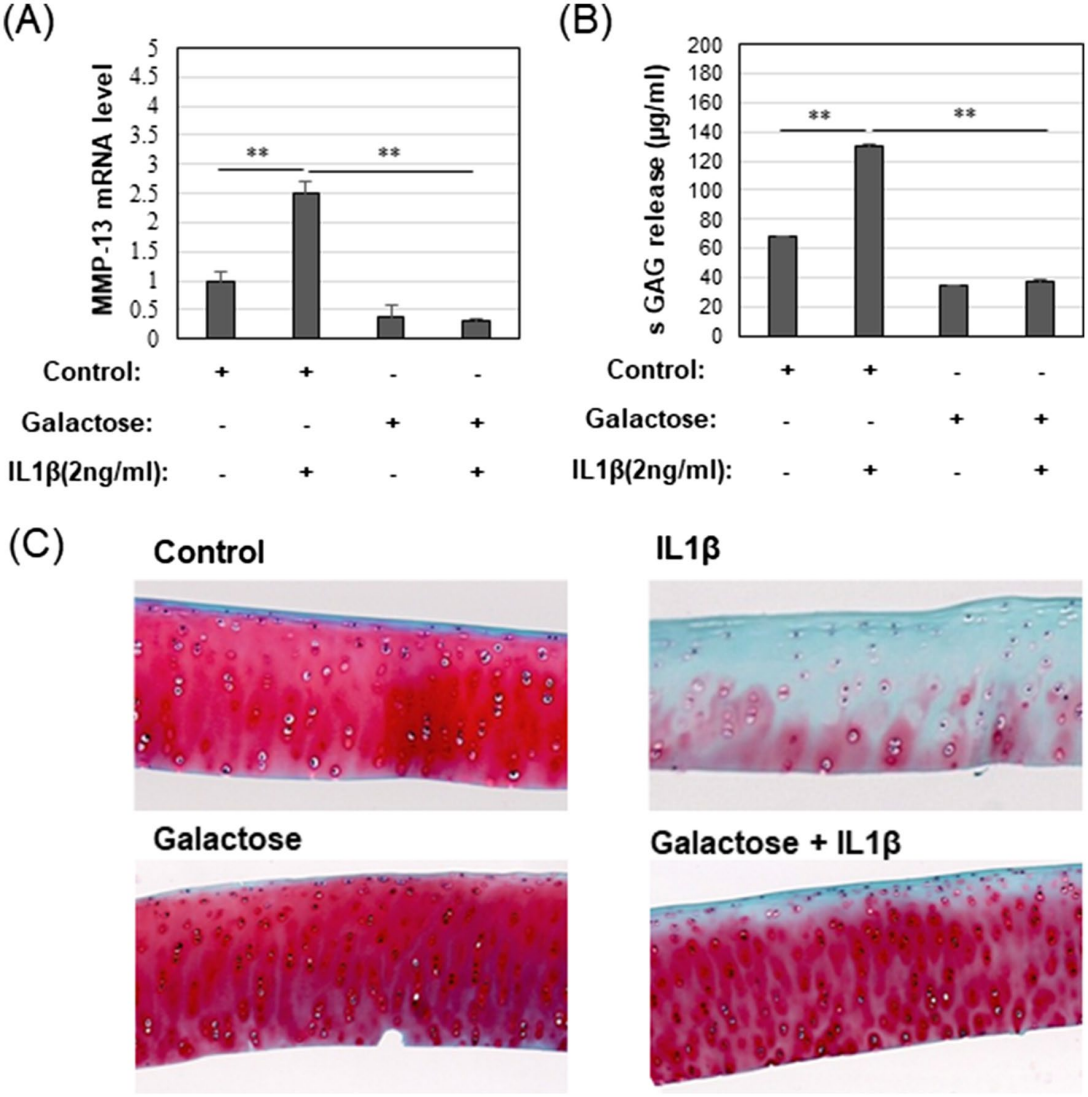
Galactose-replaced culture conditions block pro-catabolic features of activated chondrocytes and cartilage. (**A**) Expression of MMP13 mRNA of by bovine chondrocytes treated with or without IL1β in Control glucose or galactose culture medium [untreated control value set = 1.0; mean ± S.D. (error bars), n = 3]. (**B**) Quantified sulfated glycosaminoglycan released or lost from the tissue and accumulated in the medium by a colorimetric: Dimethyl-Methylene Blue (DMMB) assay (after 3 days) of bovine explant cultures [mean ± S.D. (error bars), n = 4]. (**C**) Representative images of bovine cartilage sections stained by Safranin O and Fast Green from explant cultures treated with or without 3 ng/ml IL1β in Control glucose or galactose culture medium for 7 days (n = 4). ANOVA followed by Tukey post-hoc test was used for statistical analysis. *p < 0.05; **p < 0.01**.** Another three representative images of bovine cartilage sections were shown in Supplementary Fig. S1.

Galactose appears to block damage and rescue functional integrity of mitochondria in chondrocytes treated with IL1β. To determine whether galactose-mediated rescue of mitochondria translated into effects related to OA, an examination was made of two markers of the OA phenotype, namely, expression of the collagenase MMP13 and, the loss of proteoglycan (measured as sulfated glycosaminoglycan loss) from explants of intact cartilage tissue. Chondrocytes treated with IL1β cultured in glucose-rich medium exhibited a pronounced increase in MMP13 mRNA expression as compared to control cells (Fig. 6A). When the chondrocytes were cultured in galactose-replaced medium, this increase in MMP13 mRNA was blocked (Fig. 6A). Moreover, the MMP13 mRNA levels in chondrocytes in galactose-replaced medium, with or without IL1β, were both lower than control chondrocytes cultured in glucose-rich medium.

Next, explants of intact bovine cartilage were cultured in glucose or galactose medium. After 1 week in culture, sections of control cartilage-stained bright red with safranin O, indicative of a rich proteoglycan distribution throughout the cartilage matrix (Fig. 6C). After treatment with IL1β, much of the red safranin O staining was lost from the tissue—an event that mimics the loss of proteoglycan (an important diagnostic feature) in human OA. The proteoglycan lost from the tissue can be detected in the medium by use of a colorimetric assay for sulfated glycosaminoglycan as shown in Fig. 5B. When the cartilage explants were cultured in galactose-replaced medium, no IL1β-induced loss of safranin O staining (Fig. 6C) and no increase in shed degradation products in the medium (Fig. 6B) were observed. Lower background levels of shed glycosaminoglycan were also observed in control explants (without IL1β) in galactose-replaced medium. It should also be noted that the sections of control cartilage explants grown in galactose-replaced medium appear healthy with rich safranin O staining suggesting that galactose-replacement conditions do not exert a deleterious or nutrient-deficiency effect on the tissue even after 7 days of culture.

### Effects of IL1β and galactose on human OA chondrocytes

**Figure 7:**
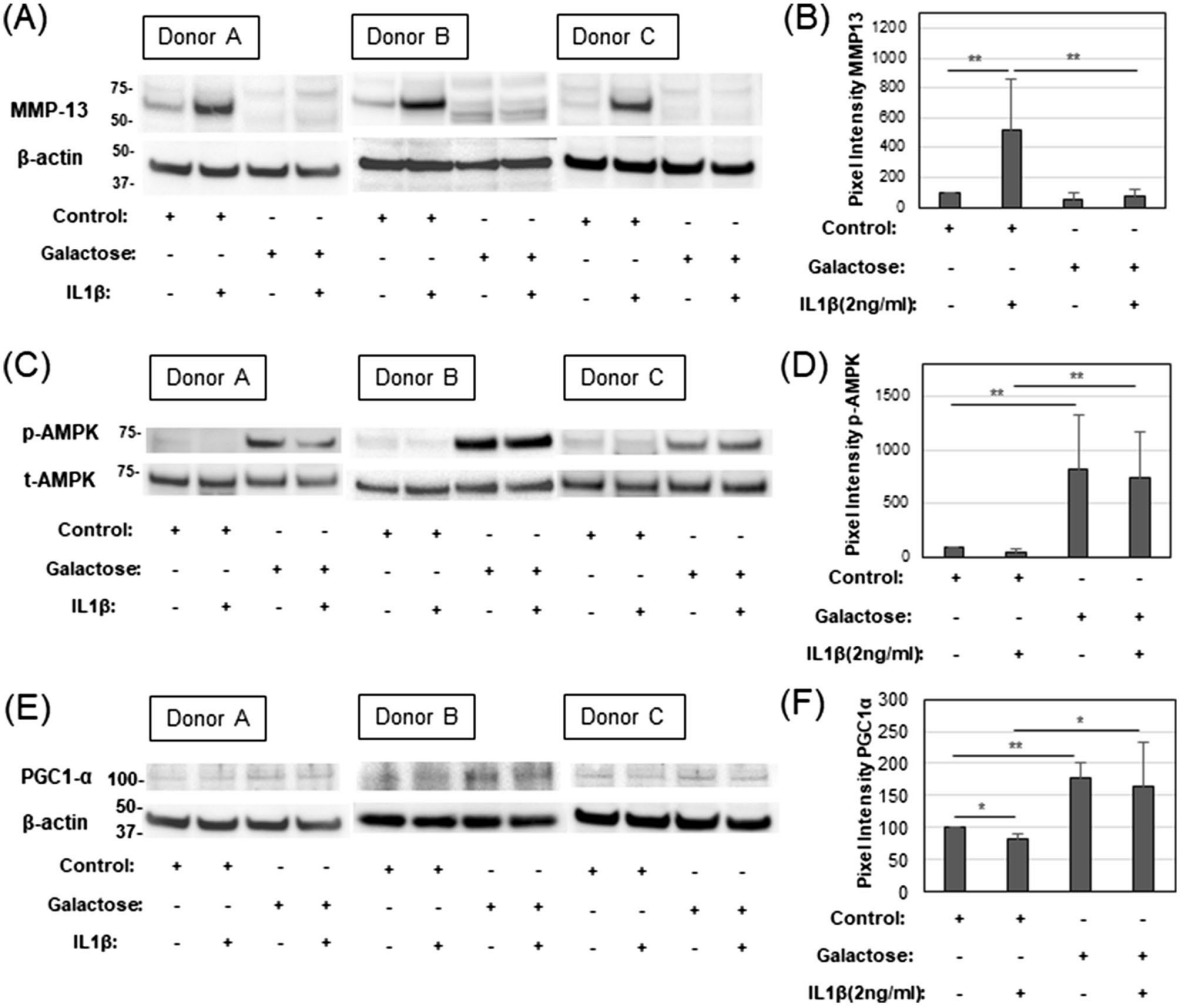
Effects of IL1β and galactose on human OA chondrocytes. Donor A is a 77 year old woman, Donor B is a 73 year old woman, Donor C is a 77 year old woman. (**A**) Representative western blots of the expression of MMP13 protein of Human chondrocytes (three different donors) treated with or without IL1β in Control glucose or galactose culture medium. (**B**) Summary of change in pixel intensity for MMP13 bands with control untreated set = 100 [mean ± S.D. (error bars), n = 3]. (**C**) Representative western blots of phospho-AMPK (p-AMPK) and total-AMPK (t-AMPK). (**D**) Summary of change in pixel intensity of bands for p-AMPKα (normalized to pixel intensity of its respective t-AMPK band) with the control, untreated chondrocytes in glucose culture medium set to 100. (**E**) Representative western blots of PGC1α and β-actin. (**F**) Summary of change in pixel intensity of bands for PGC1α (normalized to pixel intensity of its respective β-actin band) wherein the intensity of the p-AMPK band of control, untreated chondrocytes in glucose culture medium were set to 100. ANOVA followed by Tukey post-hoc test was used for statistical analysis. *p < 0.05; **p < 0.01. The entire full-length blots are shown in Supplementary Figs. S2–S4.

The effects of IL1β and galactose-replacement on mitochondrial function were next tested on high density cultures of human articular chondrocytes—cells freshly derived from human OA patient knee cartilage. For these chondrocytes, protein lysates were prepared and analyzed for the expression of key proteins related to control of mitochondrial respiration [namely, p-AMPK^12^ and PGC1α^13,14^] and MMP13, a protein marker indicative of changes in the pro-catabolic state of the cells^15^. IL1β significantly enhanced the expression of MMP13 protein in human OA chondrocytes cultured in glucose-rich medium. However, this enhancement in MMP13 protein due to IL1β was blocked in chondrocytes cultured in galactose-replaced medium. Also, background levels of MMP13 in control chondrocytes (grown in glucose medium) were reduced when the cells were grown in galactose-replaced medium (Fig. 7A,B). These results were similar to the effects of galactose on bovine MMP13 mRNA shown in Fig. 6A. Next, the same lysates were examined for expression of proteins that regulate mitochondrial function. As shown in Fig. 7C,D, control and IL1β treated human OA chondrocytes in glucose-based medium exhibited barely detectable levels of p-AMPK. However, when the control OA chondrocytes were cultured instead in medium replaced with galactose, substantially enhanced levels of p-AMPK were observed. Moreover, these enhanced p-AMPK levels were not diminished when the chondrocytes were treated with IL1β in galactose-replaced medium (Fig. 7C,D). Treatment of chondrocytes with IL1β in the presence of 2DG resulted in a similar change of IL1β-induced decrease (data not shown).

The same trend was observed in the expression of another mitochondrial regulated protein activated downstream of p-AMPK termed PGC1α (peroxisome proliferator-activated receptor-γ coactivator 1-α)^16^. Like the p-AMPK expression, enhanced PGC1α was observed in human OA chondrocytes cultured under galactose-replaced medium conditions. Again, little diminishment of PGC1α was observed following addition of IL1β in galactose-replaced medium (Fig. 7E,F).

## Discussion

In this study, we observed that chondrocytes, when cultured in a medium depleted of all glucose and replaced with galactose, underwent a critical metabolic shift. Control chondrocytes in glucose-rich medium exhibited a high dependence on the glycolysis pathway for ATP production, a dependence that became more prominent when the cells were pro-catabolically activated with IL1β treatment. This dependence on glycolysis was inversely echoed by a deficit in ATP produced through OXPHOS. As such, these conditions in glucose-rich medium mimic the mitochondrial dysfunction associated with OA^2,12,16–19^. In a galactose-replaced medium, the use of glycolysis by chondrocytes was substantially reduced; ATP produced through mitochondrial activity increased concomitant with an increase in overall mitochondrial function. More importantly, the development of pro-catabolic features following IL1β treatment, including MMP13 production and proteoglycan loss from cartilage explants, were blocked with in vitro culture in galactose-replaced media.

We used IL1β to activate chondrocytes in this study. IL1β is commonly used by many investigators to induce and mimic OA-like features, including changes in metabolism associated with OA, such as an increase in NO, leading to mitochondrial damage and release of ROS and the activation of extracellular enzymatic cartilage damage—the major issues of OA^20^. However, the genesis of human OA is more complex and multifaceted. In the natural state, the onset of OA is due to trauma, aging, inflammation, and the effects of feedback by the degradation products of tissue damage (termed DAMPs)^12,21–23^. IL1β may not induce all of the features of human disease, but it is highly reproducible, and since it is used by many investigators, it allows cross-comparisons between studies. In our previous studies, we made comparisons between IL1β and other commonly employed inducers, including; the inflammatory cytokine TNFα, three relevant examples of DAMPS, namely fibronectin fragments, HA oligosaccharides, and LPS^1,2^. Each of these inducers was capable of activating molecular markers and features of OA (such as MMP13 and proteoglycan release from cartilage) in bovine or human OA chondrocytes as well as cartilage explants.

It is known that many cells in glucose-rich culture adapt to the prominent use of the glycolysis pathway, even under aerobic conditions^24^. This has made it difficult to study the role of mitochondria and OXPHOS. In some cells, this is termed “*Crabtree Effect*”, where there is enhanced use of the glycolysis pathway for ATP production even under aerobic conditions and high external glucose concentrations and, little dependence on OXPHOS even when mitochondria are still present and functional^25^. Data in Fig. 3 of this paper suggest that functional mitochondria are present in control chondrocytes even though ~ 75% of ATP was being generated by glycolysis (Fig. 1E). To overcome these adaptive changes, investigators have used galactose-replacement. Galactose can be metabolized by the Leloir Pathway to glucose-1-phosphate and then enters the glycolysis pathway after modification to glucose-6-phosphate. However, the multi-step conversion of galactose to glucose-6-phosphate is slower than starting with glucose and results in conditions that promote oxidative phosphorylation to generate ATP^5^. Galactose can also be metabolized to glyceraldehyde-3-phosphate and pyruvate through the phosphate pentose shunt but, that process generates no net ATP and thus again forces cells to rely on mitochondrial oxidative phosphorylation for ATP^26^. Fortunately, cultured cells generally grow well under galactose-replacement conditions (Figs. 2 and S5)—conditions that allow for a more accurate evaluation of the role of mitochondria in a host of cellular activities^6,27^.

Using galactose-replacement conditions in our study, we observed that lactate production (measured as the rate of proton acidification of the medium, PER) was highly reduced; OCR (oxygen consumption related to mitochondrial respiration and OXPHOS) was very highly enhanced as compared to glucose-rich conditions (Fig. 1). These results provide evidence that control chondrocytes could switch from ~ 75% (ATP produced from glycolysis)/25% (ATP generated from OXPHOS) to 12%/88% in galactose-replaced medium (Fig. 1E). Even though the control chondrocytes were highly glycolytic, they exhibited mitochondria with an intact, functional mitochondrial membrane potential (Fig. 3). After treatment with IL-1β in glucose-rich media, the otherwise normal, healthy chondrocytes developed a pro-catabolic phenotype reminiscent of events associated with human OA. Mitochondrial function diminished (but not the number of mitochondria, Fig. 3), nitric oxide (NO), inducible nitric oxide synthase (iNOS), and reactive oxygen species (ROS) levels become elevated (Figs. 4 and 5). But critically, markers indicative of OA-like destruction of the extracellular matrix, namely MMP13 mRNA and loss of proteoglycan from cartilage explants (indicative of ADAMTS4 activity), were prominently enhanced (Fig. 6). All of these IL1β-induced features were blocked when the experiments were performed in parallel in galactose-replaced medium. Mitochondria of IL1β-treated chondrocytes in galactose-replaced medium regained membrane potential, p-AMPK levels spiked coordinate with decreases in NO, iNOS mRNA, ROS, and NMOC (Figs. 3, 4, 5, 7). These data suggest that the induced mitochondrial dysfunction and associated pro-catabolic effect in chondrocytes are reversible. The exact mechanism involved remains unknown. Gavriilidis et al. provide evidence that targeted deletion of mitochondrial superoxide dismutase-2 (SOD2) gives rise to mitochondrial dysfunction and may thus be a potent contributor to OA but also caution that there is a fine balance between anabolic and catabolic roles for SOD2^28^.

Previously we reported that HAS2-OE also blocked pro-catabolic features of activated chondrocytes, including IL1β-stimulated MMP13^1^. The biological anti-pro-catabolic effects of HAS2-OE could be mimicked via use of the chemical inhibitor, 4MU^4^. Given that 4MU acts by sequestering UDP-glucuronic acid, we postulated that the biological effects HAS2-OE effects were also due to a reduction in the intracellular pools of UDP-glucosamine and UDP-glucuronic acid. Whether chondrocytes change their phenotype when intracellular sugar pools became limiting led us to examine potential changes in overall chondrocyte metabolism. We observed that normal bovine as well as OA-derived human chondrocytes exhibited a shift toward the glycolysis and diminution of mitochondrial aerobic metabolism (as compared to controls) following treatment with IL1β, similar to the chondrocytes observed in this study using galactose-replacement media. Co-treatment with either HAS2-OE or 4MU reversed this switch in metabolic behavior to near control levels paralleled by a reversal of the IL1β-stimulated MMP synthesis^1,2,4^.

To further document that the metabolic shifts of HAS2-OE and 4MU were due, in part, to depletion of intracellular precursor pools of glucose derivatives, we examined the use at low dose of inhibitors of the glycolysis pathway such as 2DG^2^. As with HAS2-OE and 4MU, the glycolysis inhibitor 2DG rescued mitochondrial activity in activated chondrocytes, resulting in a downstream inhibition of the OA-like pro-catabolic phenotype. Additionally, HAS2-OE^1^, 4MU, or 2DG^2,4^ were able to block pro-catabolic features of chondrocytes induced by TNFα or DAMPS. However, all these experiments were performed in a standard glucose-rich culture medium.

There are several limitations to this study. As mentioned above, one limitation of this study was that chondrocytes must be grown in culture to do this kind of study, wherein they readily adapt to a high dependence on glycolysis for ATP. This is typical of many studies on metabolism that require the use of cultured cells, including many tumor cell lines^7,27,29^ and primary cultures such as myotubes^7,28^. This study, like others^7,27,29^ used the galactose-replacement approach to overcome this issue. A second limitation was limited access to human cartilage tissue. As such, studies on human OA cells were limited. It could be concluded that the data presented in Fig. 7 at least match the same trends in results shown in bovine articular chondrocytes. More work will be done in the future as samples become available. It is recognized that all of this work because it is mechanistic in nature, was performed in vitro. Thus, many questions arise, such as do these chondroprotective results (of glycolysis inhibition/OXPHOS enhancement) change due to oxygen levels, serum levels, glucose concentrations, cell density used, the timing of experiments, etc. We recently addressed this by examining the effects of 4MU in an animal (mouse) model of induced OA^30^. In this study, feeding animals 4MU provided a protective effect on the development of OA (in vivo) following medial meniscal ligament transection-induced OA. These OA protective effects were observed in vivo in the natural settings of oxygen, nutrients (e.g., high glucose and glutamine), growth factors, and loading that occur within a knee joint. This suggests that our in vitro observations of chondroprotection (by metabolic shifting) can be replicated in vivo. These data also suggest that in clinical OA within the joint, cartilage chondrocytes are likely highly dependent on glycolysis for their energy production—a metabolic feature that can be blocked by reagents such as 4MU resulting in turn, to a rescue to mitochondrial activity and a return to quiescent steady state. Also, our metabolic experiments using the 96-well plate format of the Seahorse flux analyzer were not post-normalized to cell number. However, in separate experiments (Figs. 2 and S5), we determined that our culture conditions, namely plating of chondrocytes at a high confluence density, followed by 24 h treatment with or without IL1β, 2DG, or galactose, did not affect any changes in the cell number of primary chondrocytes as measured by Hoechst 33342 dye staining analysis, direct cell counting or BCA assay for protein content of cell lysates. Thus, there was no evidence that the culture reagents or conditions exerted a toxic or proliferative effect on cultures of bovine chondrocytes that would otherwise influence the metabolic data shown in Fig. 1. Nonetheless, potential mechanical or reagent effects associated with the Seahorse flux analysis stage may have occurred. In addition to that, the verification experiments (Figs. 2 and S5) in six well plates are technically different from the actual situation in small Seahorse 96 well plates. However, we observed low well–well variation well within expected limits. Moreover, large differences between controls and IL1β, 2DG, or galactose conditions were clearly observed especially at initial baseline readout. Thus, future improvements in normalization would likely reduce our 96 well-to-well variation further; however, we expect little effect on the differences in the metabolic data between groups.

Lastly, it is unlikely that we could replicate galactose-replacement in vivo. However, that was not the goal of this study. Rather, this study demonstrated that reducing the pro-catabolic phenotype of OA may be obtained by targeting the root cause; namely fixing mitochondrial dysfunction, and that this may be a better approach than blocking cytokines or inhibiting MMPs. Even with control, human OA chondrocytes, undetectable levels of p-AMPK could be recovered by galactose-replacement, leading to inhibition of MMP13 protein production downstream. Thus, finding more promising pharmaceutical paths to generate mitochondrial reactivation in articular joint chondrocytes may lead to more effective therapeutic strategies to treat OA (or at least reduce OA progression) if used early enough in the disease. And it may not take full recovery of all mitochondria. Galactose-replacement rescued mitochondrial function better than 2DG (and 2DG matches HAS2-OE, 4MU, and DCA). However, galactose-replacement blocked increased MMP13 and cartilage breakdown to a similar extent as 2DG. Thus, forcing the activation of mitochondria, even to a small extent (as in 2DG), is sufficient for this rescue of the pro-catabolic phenotype.

## Methods

### Materials

Ham's F-12 and DMEM were obtained from Mediatech. Gibco™ DMEM, no glucose, was obtained from Thermo Fisher scientific (11966025). d-(+)-Galactose was obtained from Sigma-Aldrich (G0750). All culture medium includes 50 units/ml of Penicillin, 50 μg/ml of Streptomycin, 2 mM of l-glutamate, and 25 μg/ml of Ascorbic acid. Galactose medium means Gibco™ DMEM, no glucose mixed with 10 mM of galactose, and glucose medium means Ham's F-12 and DMEM as control. Oligomycin A (Oligomycin) was obtained from MP Biomedicals. Carbonyl cyanide p-trifluoromethoxy phenylhydrazone (FCCP), Rotenone, 2DG, and Antimycin A were obtained from Sigma-Aldrich. Pronase (53702; EMD Millipore Calbiochem), collagenase P (11249002001; Roche Applied Science), were used in the dissociation of tissues. Fetal bovine serum (FBS) was from Hyclone. IL1β was obtained from R&D Systems, Inc. Cell lysis buffer was obtained from Cell Signaling Technologies, and Clear Blue X-ray film was from Genesee Scientific. All other reagents were from Sigma–Aldrich. Hoechst 33342 was obtained from Thermo Fisher scientific (H3570). Trypsin was obtained from Sigma-Aldrich (T3049).

### Cell culture

Primary bovine articular chondrocytes were isolated from the articular cartilage of metacarpophalangeal joints of young adult steers (aged 18–24 months), which were obtained from a local slaughterhouse with approval from the North Carolina Department of Agriculture in the USA and Nagoya City Central Wholesale Market in Japan with institutional approval. No live animals were used in this study. Primary human articular chondrocytes were isolated from knee cartilage obtained from joint replacement surgery with institutional IRB approval (Nagoya University Graduate School of Medicine #2020-0146).　Document informed consent was obtained from all patients with the World Medical Association of Helsinki Ethical Principles for Medical Research Involving Human Subjects. Also, these tissues were obtained with no identifying information except age/sex. All methods were carried out in accordance with relevant guidelines and regulations. Human cartilage samples were from patients (75% female, 25% male) with an average age of 74 ± 2.2 years. Bovine and human chondrocytes were liberated from full-thickness slices of articular cartilage and cultured as described previously^1,2^. Chondrocytes were maintained using standard tissue culture procedures in a humidified incubator at 37 °C with 5% CO_2_ and atmospheric oxygen. Non-passaged primary chondrocytes were used in this study. Chondrocytes were then incubated in a serum-free medium for 1 h prior to the addition of IL1β (2 ng/ml), and/or change to galactose culture medium. Some experiments included co-treatment with 2DG (2 mM) as labeled. DMSO only at the same concentration was used as a control. Time courses varied depending on the experiment as labeled.

### Cartilage explant cultures

Full-thickness 4 mm cores of bovine articular cartilage were cultured in 1.0 ml of DMEM/Ham's F-12 medium containing 10% FBS for 48 h. The medium was then replaced with serum-free glucose and galactose culture medium, and the tissues were incubated for 4–7 days in the presence of various activators, including IL1β (3 ng/ml) and 2DG. For histology, the treated explants were fixed with 4% buffered paraformaldehyde overnight at 4 °C, rinsed in 30% sucrose, PBS, and embedded in paraffin. Sections (8 µm) were prepared and stained with safranin O for the detection of proteoglycans and counterstained with Fast Green^4^. In other experiments, the culture medium aliquots were analyzed by a colorimetric assay for released proteoglycan content by a dimethyl methylene blue assay for s-GAG release^4,31,32^.

### qRT-PCR

Total RNA was isolated from the bovine chondrocyte cultures according to the manufacturer's instructions for the use of Trizol ^R^ reagent (Thermo Fisher Scientific). It was reverse transcribed to cDNA using the iScript cDNA synthesis kit (Bio-Rad). Quantitative PCR was performed using Sso-Advanced SYBR Green Supermix (Bio-Rad) and amplified on a Step One Plus Real-Time PCR System (Applied Biosystems) to obtain cycle threshold (Ct) values for target and internal reference cDNA levels. Specific primers for real-time RT-PCR were custom-made by Integrated DNA Technologies (Coralville, IA). RT^2^ Real-Time™ SYBR Green reagents were from SA Biosciences. The bovine-specific primer sequences are as follows: MMP13^2^, forward (5′-CCT GCT GGA ATC CTG AAG AAA-3′) and reverse (5′-AGT CTG CCA GTC ACC TCT AA-3′); 18s RNA^2^, forward (5′-GTA ACC CGT TGA ACC CCA TT-3′) and reverse (5′-CCA TCC AAT CGG TAG TAG CG-3′); iNOS, forward (5′-TAC CGC ACC CGA GAT GGC-3′) and reverse (5′-TGG CAC TTC GCA CAA AGC A-3′). Real-time RT-PCR efficiency (E) was calculated as E = 10^(−1/slope)^^33^. The-fold increase in copy numbers of mRNA was calculated as a relative ratio of a target gene to 18s rRNA (ΔΔ Ct), following the mathematical model introduced by Pfaffl, as described previously^1,4^.

### Western blot analysis

Total protein was extracted using cell lysis buffer-containing protease and phosphatase inhibitor mixtures. Equivalent protein concentrations were loaded into 4–12% NuPAGE Novex Tris–acetate gradient mini-gels (Thermo Fisher Scientific). Following electrophoresis, proteins within the acrylamide gel were transferred to a nitrocellulose membrane using a Criterion blotter apparatus (Bio-Rad), and the membrane was then blocked in TBS containing 0.1% Tween 20 and 5% Bovine Serum Albumin (TBS-T-BSA) for 1 h. Immunoblots were incubated overnight with the primary antibody in TBS-T-BSA at 4 °C, rinsed three times in TBS-T, and incubated with secondary antibody in TBS-T-BSA for 1 h at room temperature. Detection of immunoreactive bands was performed using chemiluminescence (Novex ECL, Invitrogen). In some cases, the blots were stripped using Restore Plus Western Stripping Buffer (Thermo Fisher Scientific) for 30 min at room temperature and re-probed using another primary antibody. Developed X-ray films were imaged and digitized using a Bio-Rad Gel Doc with Image Lab software. Pixel intensities for MMP13 and PGC1 α bands were used for quantification after normalization to a loading control bands (β-actin). Pixel intensities for phospho-AMPKα bands were normalized by loading control bands (total-AMPK). All other experimental details not mentioned here are described in the figure legends. Specific antibodies used for analysis included rabbit polyclonal anti-MMP13 (18165-1-AP, lot 00010468, Proteintech), rabbit polyclonal anti-phospho-AMPKα^2^ (T172) (2535S, lot 10, Cell Signaling Technology), anti-total-AMPKα^2^ (D5A2) (5831S, lot 6, Cell Signaling Technology), anti-PGC1α (ab54481, lot GR3315850-1, Abcam), anti-beta-actin (4970S, lot 12, Cell Signaling Technology). Anti-rabbit IgG, HRP-linked Antibody (7074S, lot 25, Cell Signaling Technology).

### Metabolomic studies using Seahorse flux analyzer

Primary bovine chondrocytes were plated at 8.0 × 10^4^ cells/well into specially designed 96-well Seahorse XF cell culture microplates. The confluent monolayers were preincubated for 24 h with or without 2 ng/ml IL1β and with or without 2 mM 2DG in control glucose or galactose culture medium. The medium was changed to serum-free Seahorse XF Base Medium (without phenol red but with 10 mM glucose, 1.0 mM pyruvate, and 2.0 mM glutamine added) or Seahorse XF DMEM, pH 7.4, cells, depending on the assay. Assay medium also contained fresh IL1β. The cells were then mated with a sensor cartridge and analyzed in a Seahorse XFe 96 flux analyzer (Agilent Tech) for real-time detection of changes in proton accumulation and oxygen consumption following the manufacturer’s guidelines. The Seahorse Assays took advantage of five reagents: Oligomycin, FCCP, Rotenone, antimycin A, and 2DG to measure cell metabolism. Oligomycin prevents the increase in mitochondrial respiration induced by ADP without inhibiting uncoupler-stimulated respiration. FCCP is a potent uncoupler of mitochondrial oxidative phosphorylation. It disrupts ATP synthesis by transporting protons across the mitochondrial inner membrane, interfering with the proton gradient. Rotenone and antimycin A are known to inhibit complexes I and III of the electron transfer chain in the mitochondria. 2DG inhibits glycolysis by blocking hexokinase.

Briefly, for the Agilent XF cell energy phenotype test, a combined injection of oligomycin (2 μM final) and carbonyl cyanide p-trifluoromethoxy phenylhydrazone (FCCP) (0.25 μM final) were applied after the instrument completed measurement of basal values. When performing an Agilent XF Cell Mito Stress Test, timed sequential injections of oligomycin (2 μM final) followed by FCCP (0.25 μM final) and, last, a 1:1 mixture of antimycin A (0.50 μM final) with rotenone (0.50 μM final) were applied after measurement of basal values. To perform an Agilent XF Glycolytic Rate Assay, timed sequential injections of a 1:1 mixture of antimycin A with rotenone (0.50 μM final) and, last, 2DG (50 mM final) were applied after measurement of basal values. For the Agilent XF Real-Time ATP Rate Assay, timed sequential injections of oligomycin (1.5 μM final) followed by a 1:1 mixture of antimycin A with rotenone (0.50 μM final) were applied after measurement of basal values. Algorithms provided in Agilent assay report generator Excel files were used to generate blots and bar graphs. Mitochondrial ATP production derived from the Mito Stress test is expressed as OCR (pmol of O_2_/min); in the software, ATP production in these OCR units is closely proportional to true ATP values. A measure of ATP production rate in terms of pmol/min ATP is provided by the ATP rate assay. We determined that our culture conditions, namely plating of chondrocytes at a high confluence density in 6-well culture plates, followed by 24 h treatment with or without IL1β, 2DG, or galactose, did not affect any changes in the cell number of primary chondrocytes as measured by Hoechst 33342 dye staining analysis, direct cell counting or BSA assay for protein content of lysates (Figs. 2 and S5). As such, no additional normalization of cell counts was taken at the end of Seahorse analyses.

### Testing the effects of culture conditions on chondrocytes

3.0 × 10^6^ cells primary bovine articular chondrocytes were cultured on six well plates (Techno Plastic Products) with the same culture plan as other experiments in this study. Chondrocytes were incubated in DMEM, added IL1β (2 ng/ml), 2DG (2 mM), and/or change to Galactose culture medium for 24 h. DMSO only at the same concentration was used as a control. 2 ml of culture medium was used for each well. *Direct cell counting* Chondrocytes were rinsed with sterile PBS, then were collected by 1 ml of Trypsin. Chondrocytes were counted by an automated cell counter (BIO-RAD, TC20™) after adding 500 μl of DMEM. *Protein quantification* Chondrocytes were rinsed with sterile PBS, then protein was extracted using cell lysis buffer–containing protease and phosphatase inhibitor mixtures. BCA Protein Assay Kit (23225, Thermo Fisher Scientific) was used for protein quantification. *The fluorescence staining of the nucleus* Chondrocytes were rinsed with sterile PBS, then stained by Hoechst 33342 following the product information. Chondrocytes were observed by a fluorescence microscope (BZ-X800, Keyence), and the total cell occupied area and color intensity were quantified by an analysis software (Hybrid cell count, Keyence).

### Mitochondria staining

MitoPT TMRM Assay (TMRM) (Catalog#: 9105, Immunochemistry Technologies) (10 μM final) and Mito Tracker™ Green FM (Mito Green) (Catalog#: M7514, Thermo Fisher Scientific) (200 nM final) were used following the product information sheet. TMRM, which is a mitochondrial dye reagent having a delocalized positive charge, accumulates in an active, negatively charged mitochondrial membrane, and stains healthy mitochondria red. When the mitochondrial membrane potential collapses, TMRM becomes distributed throughout the cytosol. Mito Green stains all mitochondria green by reacting with the free thiol groups of cysteine residues belonging to mitochondrial proteins. Mitochondria were observed by a fluorescence microscope (BZ-X800, Keyence). The color intensity was quantified by an analysis software (Hybrid cell count, Keyence). Mito Green estimated the total number of all mitochondria; TMRM estimated the number of active mitochondria. TMRM/Mito Green Ratio, which is calculated by dividing the red color intensity of TMRM by the green color intensity of Mito Green is used for evaluation.

### Nitric oxide assay

The culture media of some conditions were collected after 24 h cell culture. The amount of Nitrite and Nitrate in the cell culture medium was measured by Greiss reagents^34^: Nitric Oxide Assay Kit (Catalog#: EMSNO, Invitrogen) following the product information sheet. The color change was quantified by absorbance, then the amounts calculated by using a standard curve. Levels of Nitric Oxide generated were defined as the total of Nitrite and Nitrate detected^35^.

### Reactive oxygen species assay

Intracellular Reactive Oxygen Species (ROS) were stained by the Cellular ROS Assay Kit (Catalog#: ab113851, Abcam) following the product information sheet after 24 h of cell culture. It includes the cell-permeant reagent; 2′,7′-dichlorofluorescein diacetate (DCFDA), which stains intracellular ROS green. Stained-cells were observed by a fluorescence microscope (BZ-X800, Keyence), and the color intensity was quantified by an analysis software (Hybrid cell count, Keyence).

### Statistical analysis

Values are expressed as mean ± standard deviation (SD). One-way ANOVA followed by Tukey post-hoc tests are used for multiple-group comparisons and a two-tailed unpaired Student's t-test was used for direct comparison of the treatment group with control. A p-value of < 0.05 was considered significant. *p < 0.05; **p < 0.01. Those statistics were performed by EZR^36^.

## Supplementary Information

Supplementary Figures.

## Data Availability

All data generated or analyzed during this study are included in this published article.
