# Supplementary material for: Metabolic reprogramming in chondrocytes to promote mitochondrial respiration reduces downstream features of osteoarthritis
Source: Sci Rep. 2021 Jul 23;11:15131. doi: 10.1038/s41598-021-94611-9 (PMC8302637; doi:10.1038/s41598-021-94611-9)

Metabolic reprogramming in chondrocytes to promote mitochondrial respiration reduces downstream features of osteoarthritis

Author names and affiliations

Yoshifumi Ohashi[1], Nobunori Takahashi[1*], Kenya Terabe[1*], Saho Tsuchiya[2], Toshihisa Kojima[1], Cheryl B. Knudson[3], Warren Knudson[3], and Shiro Imagama[1]

1. Department of Orthopedic Surgery, Nagoya University Graduate School of Medicine, 65 Tsurumai-cho, Showa-ku, Nagoya 466-8550, Japan.

2. Department of Orthopedic Surgery, Japan Community Health care Organization, Tokyo Shinjuku Medical Center, 5-1 Tsukudo-cho, Shinjuku-ku, Tokyo 1628543.

3. Department of Anatomy and Cell Biology, Brody School of Medicine, East Carolina University, Greenville, North Carolina 27834

Supplementary Figure S1:
The other three Representative images of bovine cartilage sections

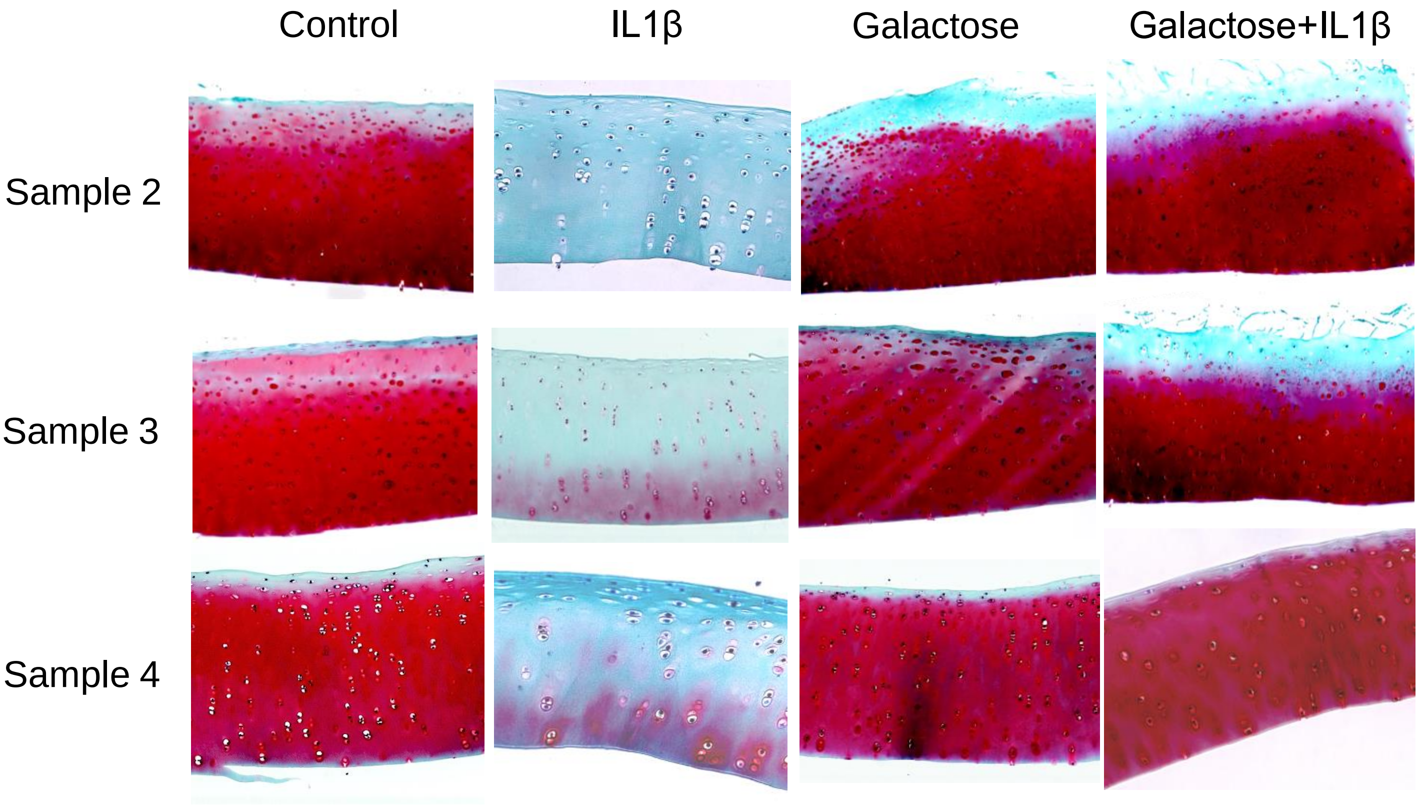

Supplementary Figure S2: Entire blot of Fig 7 (A)

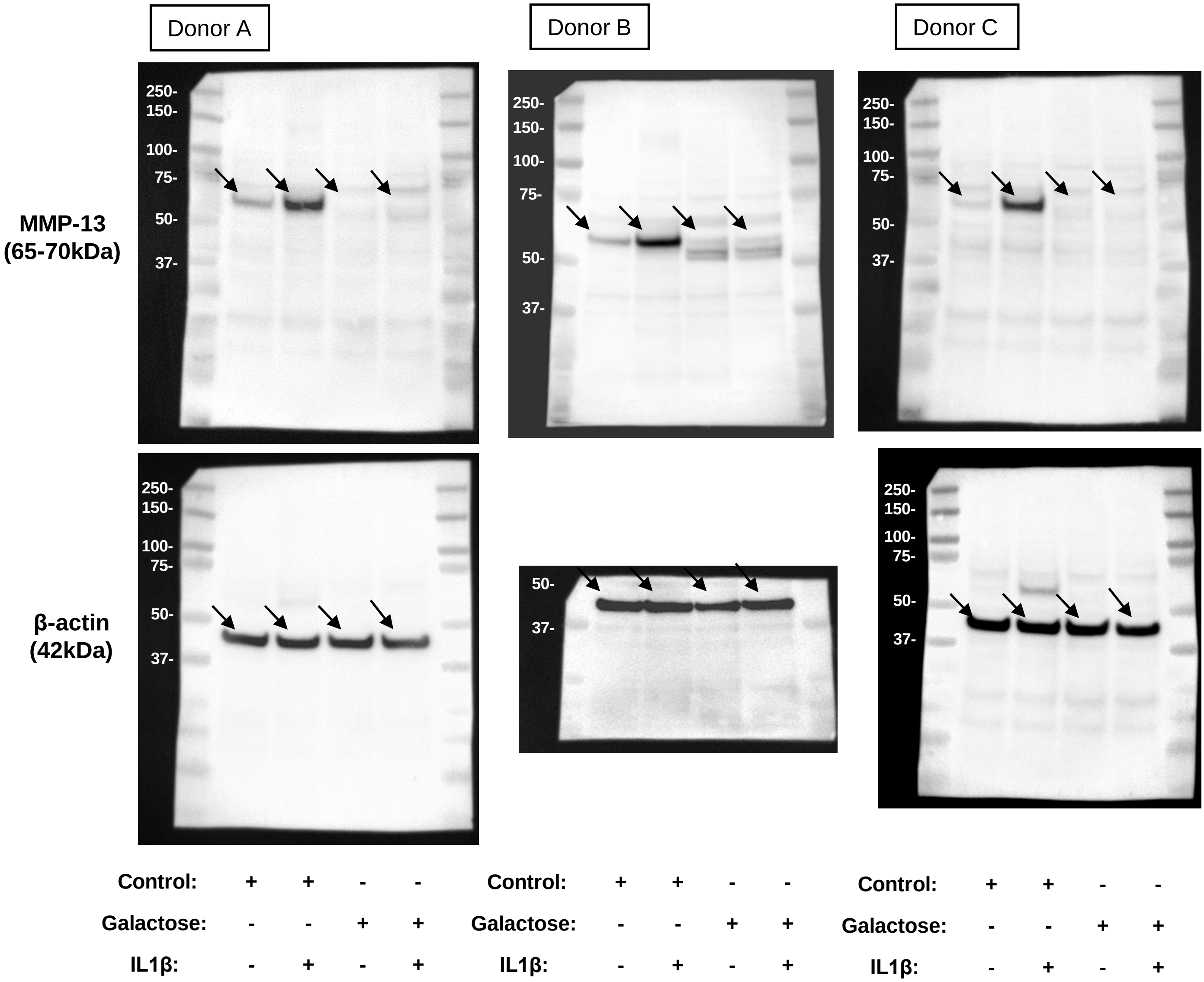

Supplementary Figure S3: Entire blot of Fig 7 (C)

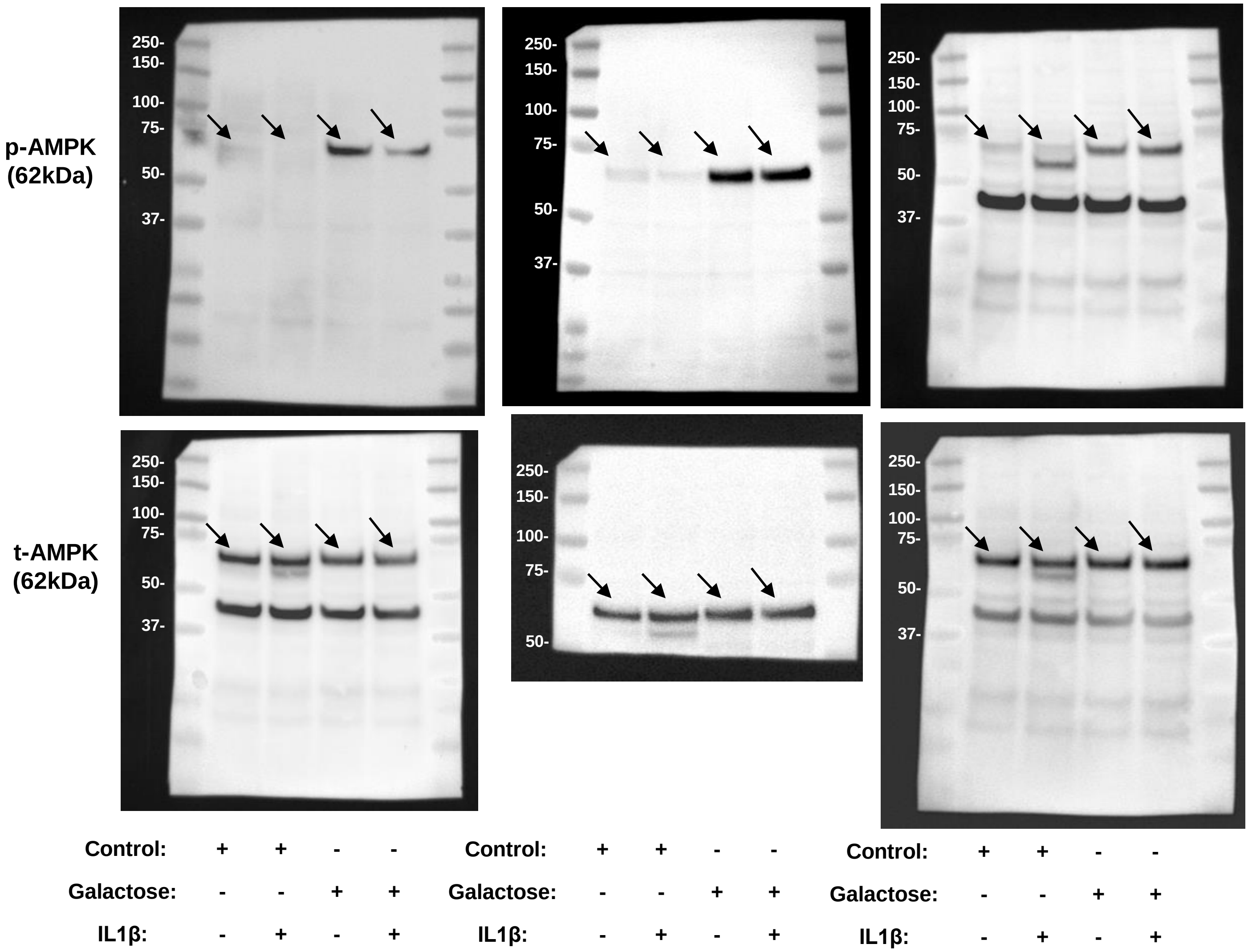

Supplementary Figure S4: Entire blot of Fig 7 (E)

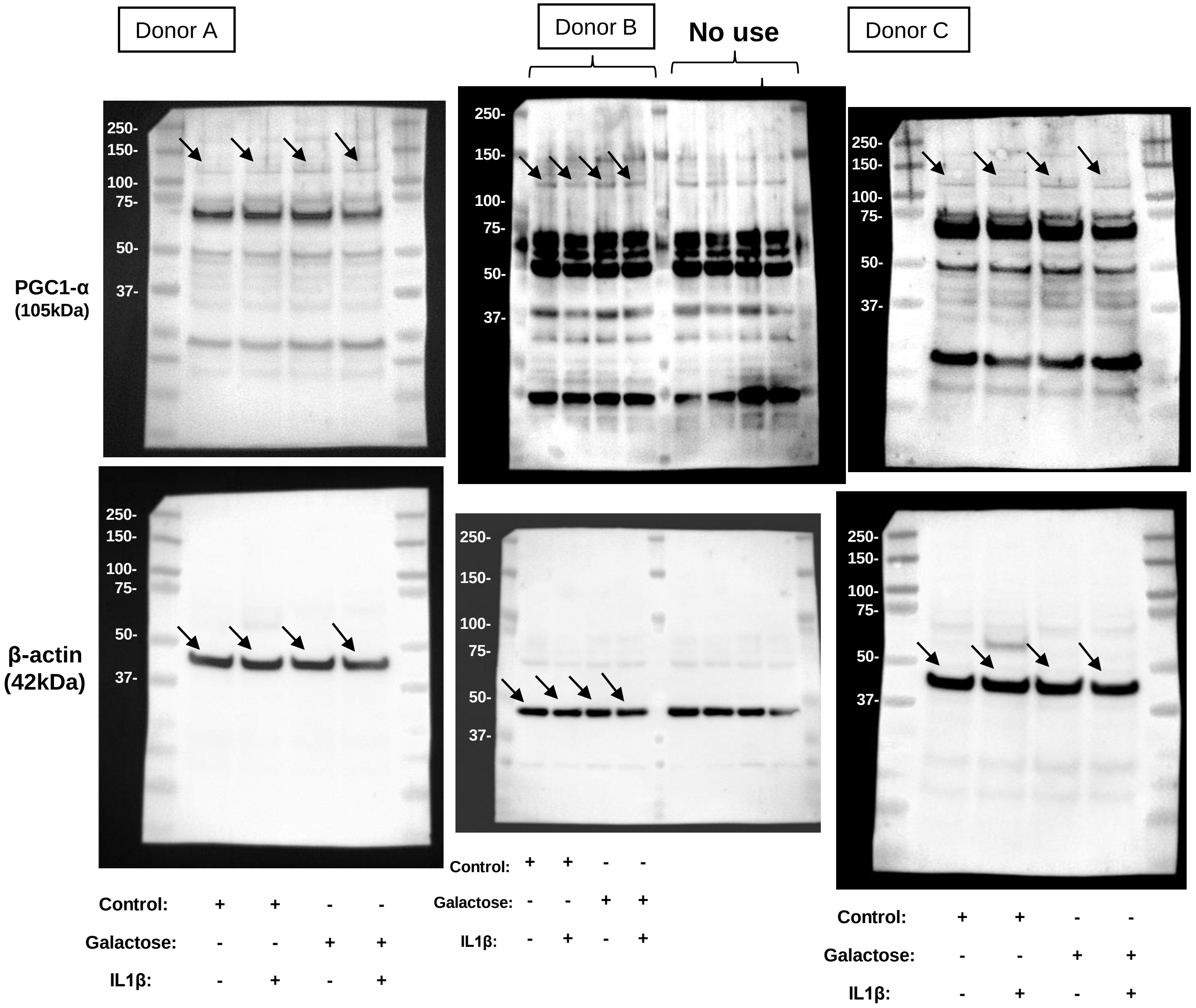

Supplementary Figure S5: Representative images of Fig 2

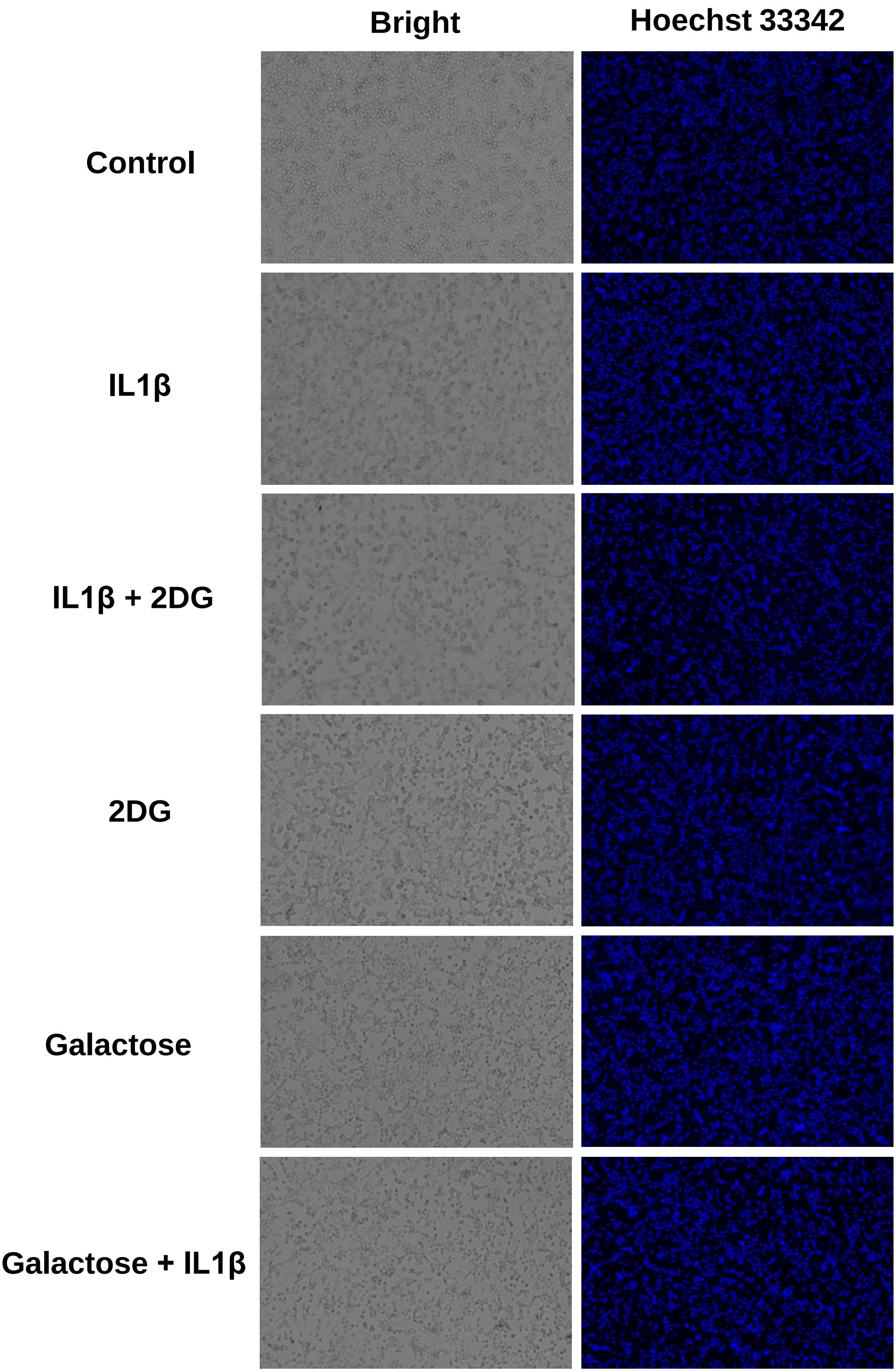

Supplement: Supplementary file 1 — Supplementary Figures. [file 41598_2021_94611_MOESM1_ESM.pdf]
